# Hypoglossal nerve stimulation in adolescents with down syndrome and obstructive sleep apnea: A systematic review and meta-analysis

**DOI:** 10.3389/fneur.2022.1037926

**Published:** 2022-10-25

**Authors:** Pan Liu, Weiguo Kong, Caijing Fang, Kangxu Zhu, Xiaohua Dai, Xiangming Meng

**Affiliations:** ^1^Department of Emergency or ICU, Anhui Provincial Hospital of Integrated Traditional and Western Medicine, Hefei, China; ^2^Graduate School of Anhui University of Traditional Chinese Medicine, Hefei, China; ^3^Department of Otolaryngology, Wuxi Huishan District People's Hospital, Wuxi, China; ^4^Branch Center of the National Clinical Research Center for Cardiovascular Disease, The First Affiliated Hospital of Anhui University of Traditional Chinese Medicine, Cardiovascular Institute of Anhui Academy of Chinese Medicine, Hefei, China

**Keywords:** hypoglossal nerve stimulation, down syndrome, obstructive sleep apnea, adolescents, apnea-hypopnea index

## Abstract

**Objective:**

To evaluate the efficacy and adverse effects of hypoglossal nerve stimulation in adolescents with down syndrome and obstructive sleep apnea.

**Methods:**

A systematic search was conducted using PubMed, Web of Science, Embase, and Scopus databases. The systematic review followed the Preferred Reporting Items for Systematic Reviews and Meta-Analyses guidelines. A comprehensive search strategy used a combination of Medical Subject Headings and free words with “OR” and “AND.” Articles were screened to extract data reporting apnea-hypopnea index, quality of life, voltage, follow-up duration, and complications. All included participants were adolescents with down syndrome and obstructive sleep apnea.

**Results:**

A total of 92 articles were identified, of which 9 articles met the inclusion criteria. A total of 106 patients were included. All the studies showed that patients receiving hypoglossal nerve stimulation experienced a significant decrease in apnea-hypopnea index (at least 50%). The pooled AHI was significantly lower in patients following treatment (mean AHI reduction 17.43 events/h, 95% confidence interval 13.98–20.88 events/h, *P* < 0.001) after 2 case reports were excluded. The pooled OSA-18 were significantly decreased in 88 patients after treatment (mean OSA-18 reduction 1.67, 95% confidence interval 1.27–2.08, *P* < 0.001) after excluding 5 studies. Four investigations examined the necessity to optimize stimulation voltage for arousal during treatment. The most common complication was pain or discomfort in the tongue or mouth. Most studies had relatively short patient follow-up periods, with the most extended follow-up being 44–58 months.

**Conclusion:**

Hypoglossal nerve stimulation significantly reduces apnea-hypopnea index and improves the quality of life; and thus, could be a potential alternative therapy for obstructive sleep apnea in adolescents with down syndrome. The adolescent's age, potential complications, adverse events, long-term efficacy, and comfort, needs to be considered while performing hypoglossal nerve stimulation.

## Introduction

Obstructive sleep apnea (OSA) is a sleep-related breathing disorder that causes hypoxia and fragmented sleep, because of repeated airway obstruction or collapse (1). OSA affects approximately 5% of healthy children globally and is associated with concomitant behavioral issues, such as inattention, hyperactivity, and/or cognitive decline in the pediatric population (2). Obesity and craniofacial deformities are the most common causes of airway obstruction (3).

Down syndrome (DS), trisomy 21, or redundancy of chromosome 21, is one of the most complex human congenital diseases (4). Adolescents with DS show several unique characteristics, such as generalized hypotonia, macroglossia, facial hypoplasia, small tracheal caliber, and lingual tonsillar hypertrophy (5). Up to 80% of children with DS have OSA, which is thought to be caused by these unique characteristics (6).

Untreated OSA can affect a child's development, including reduced learning abilities, speech and language delays, and impaired cognitive flexibility and memory (7). Currently, upper airway surgery and continuous positive airway pressure (CPAP) are the commonly used treatments for OSA (8). Although for most individuals OSA improves after treatment, the incidence of residual airway obstruction remains high (9). Following upper airway surgery, residual airway obstruction can cause up to 75% of children to require breathing support (10). Furthermore, compliance with CPAP is not good enough to meet treatment needs due to discomfort, inconvenience, and cognitive delay (11).

Since 2014, hypoglossal nerve stimulation (HNS) has been approved by the US Food and Drug Administration (FDA) for treating OSA in adults (12). HNS improves breathing while sleeping, by stimulating the muscles in the upper airway and by hardening the tongue and soft tissues (13). Studies have shown that HNS is more tolerable and less irritating than CPAP and upper airway surgery, and it is an effective treatment for moderate-to-severe OSA in adolescent patients (14).

However, there is no consensus about reducing OSA in adolescents with DS using HNS. In 2016, the first case of HNS for OSA in adolescents with DS was reported (15). Although most research in recent years have shown that HNS is a better option, these studies have limitations in terms of sample size, in follow-up duration, and in documentation of complications (16).

To the best of our knowledge, this is the first review of HNS for treating OSA in adolescents with DS based on the existing research. We sought to evaluate the efficacy and adverse effects of HNS in adolescents with DS and OSA and to clarify the underlying processes of HNS for treating OSA.

## Methods

This systematic review was conducted following the Preferred Reporting Items for Systematic reviews and Meta-Analyses guidelines (17). Ethical approval is not required for this review.

### Search strategy

A systematic search was carried out through PubMed, Web of Science, Embase, and Scopus databases. The search strategy used a combination of Medical Subject Headings (MeSH) and free words with “OR” and “AND.” Retrieval words included “DS,” “Trisomy 21,” “OSA,” “sleep apnea syndromes,” “HNS,” and “upper airway stimulation.” The detailed retrieval strategy is available in Supplementary material. The final literature search was completed on June 25, 2022. Two reviewers (WK and KZ) independently qualified the studies and extracted the data. Differences were resolved through discussion between the two reviewers.

### Inclusion and exclusion criteria for study selection

The requirements for studies to be included were as follows: (I) Types of participants – adolescents (the age ranges from 10 to 21 years) (18) with DS and OSA; (II) type of intervention - HNS; and (III) type of language - English. The exclusion criteria included relevant publications, reporting of only surgeries, cell experiments, comments, no outcomes, not adolescents, and repeat investigations.

### Data extraction

Two reviewers (WK and KZ) extracted data using Excel (Microsoft Inc., USA) spreadsheet. The data included were as follows: authors, year of publication, study design, voltage titration, sample size, age, treatment assessment, intervention time, follow-up duration, main results, adverse events, and conclusions.

### Assessment of risk of bias

Two reviewers (WK and KZ) independently evaluated the selected articles. The quality of the articles included in this review was assessed using the National Institutes of Health quality assessment tools (observational cohort studies and cross-sectional studies) (19). The quality of each article was rated as “good,” “fair,” or “poor” according to its overall quality score. Any disagreement was resolved by consensus through discussions between the two reviewers.

### Statistical analysis

Mean differences (MD) were calculated to create forest plots of continuous data to analyze the variations in the apnea-hypopnea index (AHI) and A validated, disease-specific quality of life instrument for OSA (OSA-18) between HNS and non-HNS. We merged data using Review Manager 5.3 software. The test was regarded as statistically significant when the *P* value was <0.05 and 95% confidence intervals (CIs) were given. The Q statistic, with a significance level of *P* < 0.10, was used to investigate the heterogeneity of Mean differences. The study was divided into two groups according to whether the number of cases was >10, and a subgroup analysis was conducted. The random-effects model was employed throughout the analysis.

## Results

### Search outcome

A total of 92 articles were obtained from the 4 databases, out of which 41 articles remained after excluding the duplicate ones. The literature was then further screened using the article titles and abstracts and 20 irrelevant articles were excluded. Next, two abstracts without full text, one surgical procedure study, one with cellular experimental study, four review articles, one no result article, and three non-adolescent studies, were also excluded by full-text literature identification. Ultimately, 9 studies met the inclusion criteria for this systematic review.

**Figure 1:**
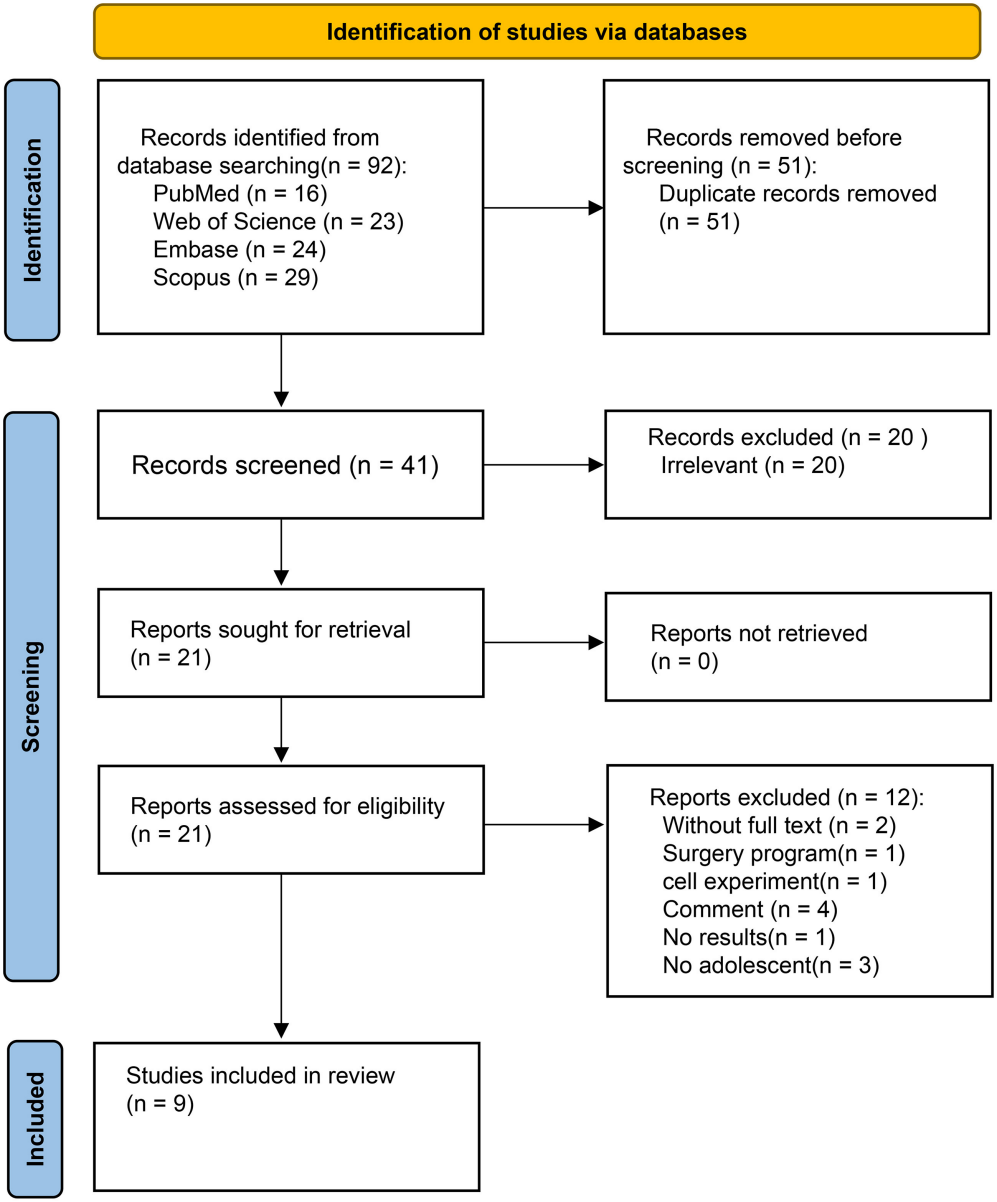
Flow chart showing the process of literature screening.

A total of 106 patients were included in all investigations; three articles had a sample size with more than 10, while 6 articles had a sample size of no more than 10 cases. The first related publication was published in 2016. Further, an article was published each, in 2017, 2019, and 2020 years. Because of the rising interest in this topic, four relevant articles were published in 2021. Table 1 summarizes the studies included in the review. The flow chart of the literature search is shown in Figure 1.

**Table 1:** A summary of studies on hypoglossal nerve stimulation for adolescents who had down syndrome and OSA.

**References**	**Study design**	**Cases (*n*)**	**Age**	**Treatment assessment**	**Intervention time (h)**	**Voltage titration** **(v)**	**Follow-up duration (months)**	**Main results**	**Adverse events**	**Conclusions**	**Risk of bias**
Yu et al. (18)	Prospective single-group multicenter cohort study	42	15.1 ± 3.0	PSG, ESS, OSA-18	9.0 ± 1.8/night	Changed but no more than 1.0	12	AHI was decrease (*P* < 0.05); OSA-18 and ESS score was improved (*P* < 0.05); the most common complication was temporary oral discomfort (11.9%).	Yes	HNS is able to be safely performed.	Good
Kay et al. (20)	Case report	1	13	HSAT, PSG, CAI, CO2%	10.5/night	1.6,1.9,2.0	8	8 months after surgery: sleep efficiency improved; AHI decreased from 44.9 to 12.2; min CO2%:90%.	No	HNS is effective in reducing OSA burden.	Poor
Yu et al. (21)	Prospective longitudinal, multicenter single-arm trial	20	15.5	PSG, OSA-18,ESS	NA	Unclear	12	The mean decrease in AHI was15.1 (*P* < 0.001); OSA-18 and the ESS score was lower.	No	HNS treatment is safe and effective	Good
Grieco et al. (22)	Prospective study	9	15.2 ± 3.4	PSG; Neurocognitive and behavioral testing	Unclear	Unclear	6.5	There was a significant mean decrease in AHI by 11.0 (*P* < 0.05); all neurocognitive and behavioral testing scores are improved.	No	The benefits are reduced AHI and improved some neurocognitive and behavioral outcomes.	Fair
Stenerson et al. (23)	Case series	4	10–13	PSG; OSA-18	Unclear	1.5, 1.8, 1.9, 2.1,2.2, 2.8	44–58	AHI decreased by at least 50% in all participants; OSA-18 scores improved in 3 participants; but 2 participants exhibited severe OSA when the device was turned off.	No	HNS effectively controls their OSA, but their underlying untitrated OSA appears to persist into adulthood.	Good
Karlik et al. (24)	Case series	3	10–19	PSG; Anesthetic and medications	Unclear	No	Unclear	The average AHI change was 87.4%; tailored anesthesia protocols improve patient outcomes.	No	HNS combined with individualized perioperative management can improve OSA symptoms in patients	Good
Caloway et al. (25)	Case series	20	The median 13.75–17.25	PSG, OSA-18	The median 9.21/night	Unclear	2	Median percent reduction in AHI of 85%; The median OSA-18 score	Yes	HNS treatment effectively reduced	Good
								reduction was 1.15		the patient's AHI score and improved quality of life.	
Diercks et al. (26)	Case report	6	12–18	PSG; OSA-18	5.6–10/night	No	6–12	Demonstrated a 56% to 85% reduction in AHI; the total score for OSA-18 improved (1.5 ± 0.6).	Yes	HNS is a potential therapeutic option.	Good
Diercks et al. (15)	Case report	1	14	PSG	8–9/night	1.3,1.4,1.5	5	Baseline of AHI was 48.5; with increasing stimulation voltage (1.1–1.5v), AHI decreased (10.6–3.4 events/h).	No	HNS is well tolerated and significantly improved OSA.	Fair

HGS, Hypoglossal nerve stimulation; AHI, Apnea-hypopnea index; PSG, Polysomnographic; ESS, Epworth Sleepiness Scale; OSA-18, A validated, disease-specific quality of life instrument for OSA; HSAT, Wrist-based home sleep apnea test; CAI, central apnea index; CO2%, Oxygen saturation; ODI, Oxygen desaturation index.

### Treatment outcomes

#### AHI

**Figure 2:**
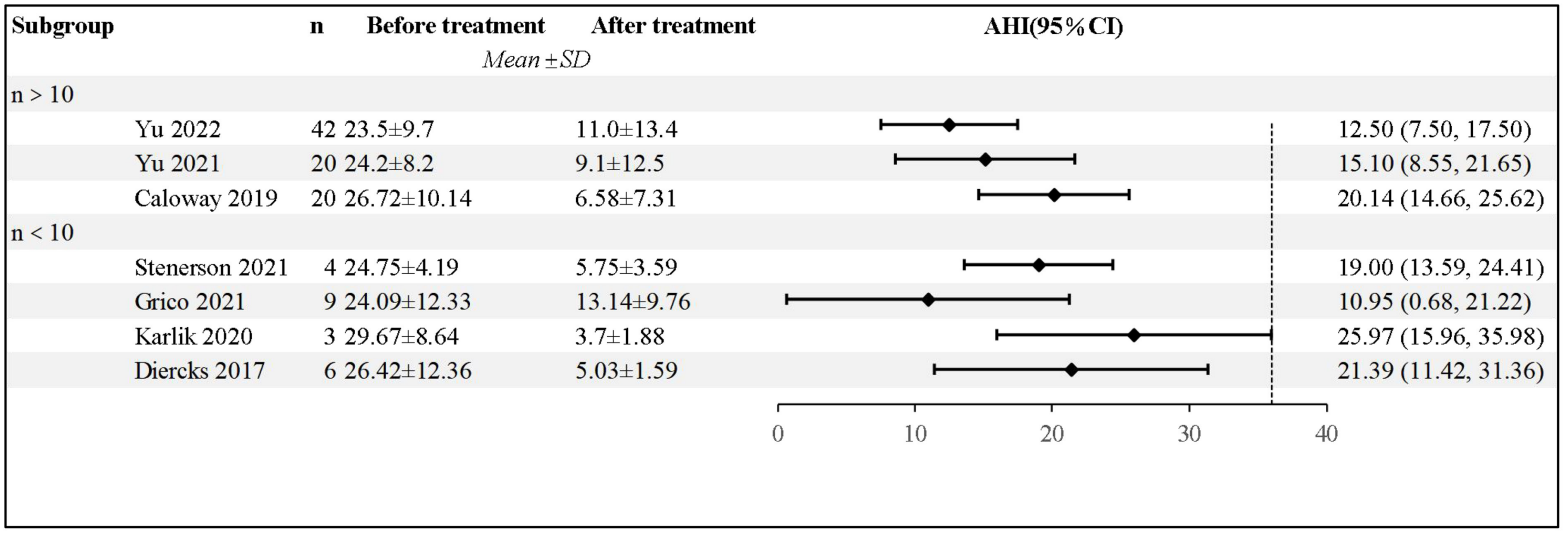
The forest plot of studies investigating AHI.

All the studies showed that patients receiving HNS experienced a significant decrease in AHI, based on the Polysomnography (PSG) results. Diercks et al. reported the first case of HNS treatment for a 14-year-old boy, whose AHI dropped from 48.5 to 3.4 events/h (15). Yu et al. followed 42 patients for a year; the findings revealed that the average AHI decreased by 12.9 ± 13.2 events/h, 65.9% of the patients experienced a 50% decrease in AHI, and 73.2% of the patients had an AHI of <10 events/h (18). After excluding 2 case reports, a total of 104 patients were included in the analysis; pooled data revealed significantly lower AHI in patients after HNS (mean AHI reduction 17.43 events/h, 95% CI 13.98–20.88 events/h, *P* < 0.001); however, there was moderate heterogeneity between the studies (I^2^ = 42%, *P* =0.11). The forest plot of studies investigating AHI is shown in Figure 2.

#### Quality of life

**Figure 3:**
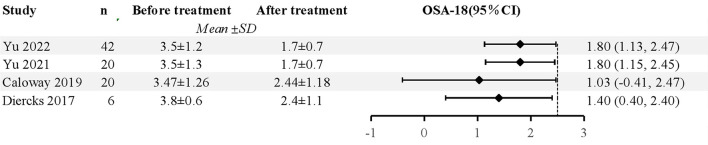
The forest plot of studies investigating OSA-18.

Five studies used the OSA-18 (a validated, disease-specific quality of life instrument for OSA) and Epworth Sleepiness Scale (ESS) questionnaires, to examine the improvements in treatment durations (18, 21, 23, 25, 26). As a result, it improved their sleep quality and subjective feelings. Kay et al. found that snoring, daytime sleepiness, behavioral problems, and supine sleeping improved in these patients (20). According to two studies, HNS can decrease patients' ESS scores in terms of sleep quality (18, 21). In addition, Greco et al. found that participants' neurocognitive and behavioral outcomes were also amended (22). A total of 88 patients were included after 5 studies were excluded, and the pooled data revealed significantly decreased OSA-18 in patients following HNS (mean OSA-18 reduction 1.67, 95% CI 1.27 to 2.08, *P* < 0.001), and there was no evidence of study heterogeneity (I^2^ = 0%, *P* = 0.43). The forest plot of studies investigating OSA-18 is shown in Figure 3.

#### Voltage

Four investigations examined the necessity to optimize stimulation voltage for arousal during treatment (15, 18, 20, 23). According to research, AHI decreases as stimulation voltage increases, with 21.1 events/h at 1.3 V, 3.7 at 1.4 V, and 3.4 at 1.5 V (15). The remaining five investigations, however, did not specifically record any information regarding the titration voltage (21, 22, 24–26).

#### Follow-up duration

Most researchers followed patients with DS and OSA for a short duration. Only one study had a follow-up duration of 44 to 58 months (23), two studies had <6 months (15, 25), five studies had 6 to 12 months (18, 20–22, 26), and one study provided no details regarding follow-up duration (24). Noticeably, two participants had a persistently moderate OSA after 44–58 months of follow-up, postoperatively (23).

#### BMI

Eight studies stated that HNS treatment should consider the influence of body mass index (BMI) factors (15, 18, 20, 21, 23–26), and five advised that patients should have a BMI of <32 kg/m^2^ (15, 18, 21, 23, 26). However, only two studies performed BMI data analysis. There were 11 patients with BMI in the 85th percentile or greater. Five (45.5%) of these 11 patients responded to therapy, compared to 44.4% of patients with a BMI under the 85th percentile (*P* = 0.96) (21). Age- and sex-adjusted BMIs ranged from 19.2 to 24.6 kg/m^2^ at baseline, and from 19.8 to 34.6 kg/m^2^, and BMI percentiles increased for 3 of the four patients (23).

#### Complications

**Figure 4:**
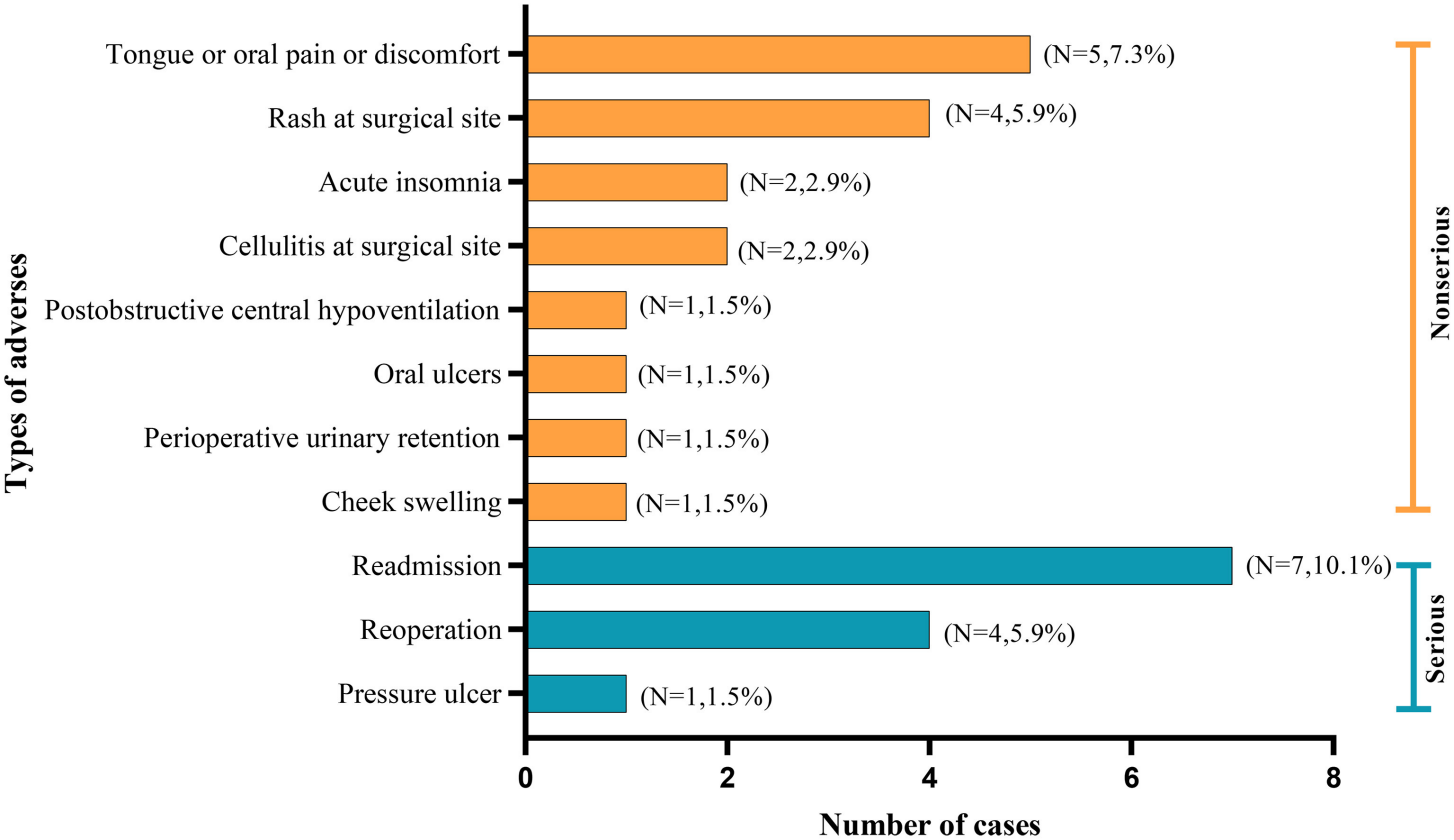
The distribution of complications of studies included in the systematic review.

Pain or discomfort in the tongue or oral cavity, were the most common complications. Notably, three studies documented the occurrence of serious adverse events, such as the incidences of reading and reoperations (18, 25, 26). The distribution of complications is shown in Figure 4.

## Discussion

In this review, we investigated the therapeutic effects of HNS for OSA in adolescents with DS, the enhancement of participants' quality of life, and the benefits of the intervention, based on the current literature. In addition, we discussed its potential therapeutic mechanism. After receiving HNS therapy, all participants experienced a significant reduction in AHI. Participant's OSA-18 and ESS scores also indicated noticeable improvements in some studies. Therefore, HNS can be considered as an effective treatment for OSA in adolescents with DS and is a better tolerated option than CPAP or equally well tolerated option than CPAP.

OSA is a complex condition that demands a multimodal treatment strategy, particularly for adolescents with DS, due to the abnormalities in their airway structure (27). Nowadays, tonsillectomy, adenoidectomy, and CPAP are reliable options used to alleviate OSA (28). However, even after surgery or after CPAP use is discontinued, the condition can persist (29). Previous studies have proven that HNS is an effective treatment option for OSA in adults, particularly for those who cannot tolerate CPAP therapy (30). According to a single-center study, the mean AHI for patients with OSA who were treated with HNS decreased from 38.9 ± 12.5 to 4.5 ± 4.8, whereas the mean AHI for those who underwent uvulopalatopharyngoplasty surgery decreased from 40.3 ± 12.4 to 28.8 ± 25.4 (31). Consequently, HNS appears to have a more favorable therapeutic outcome than uvulopalatopharyngoplasty surgery for patients with OSA.

With the advancement of medical technology, electrical stimulation devices have shown numerous benefits in treating OSA. According to research, the loss of genioglossal muscle tone is strongly correlated with airway collapse (32). A hypoglossal nerve stimulator comprises of an implantable pulse generator (IPG), a pressure sensor to detect breathing, and a stimulation lead connected to the sublingual nerve (33). The pressure sensor monitors chest wall motion, allowing the IPG to signal the end of expiration and the beginning of inspiration. The stimulation is subsequently delivered to the hypoglossal nerve through the IPG, and the stimulation leads can specifically activate certain branches of the hypoglossal nerve, which enhances the stiffness and protrusion of the tongue (34). Tongue protrusion expands the cross-sectional dimensions of the airway, consequently facilitating the patient's airway; and thus, preventing airway collapse (35).

The degree of AHI reduction and quality of life improvement, are the essential measures to monitor the effectiveness of therapeutic modalities for treating OSA. All the adolescents included in this systematic review had used CPAP before receiving HNS therapy, but none of them was able to tolerate it. The PSG of all the participants showed a significantly lower AHI score during the HNS therapy, than that before receiving it. This result is comparable to that obtained using typical pediatric CPAP treatment. According to King et al., CPAP therapy decreased the AHI of pediatric patients from 9.8 (5.7–46.0) to 3.3 (0.4–2.2) (36). Interestingly, one study found that discontinuing HNS did not immediately revert patients to their initial AHI level (23). In addition, employing the ESS and OSA-18 questionnaires, it was determined that the quality of life of these patients improved after receiving HNS treatment (18, 21, 23, 25, 26).

Although there are positive findings on the efficacy of HNS in adults, the data are inconsistent. Zhu et al. followed 82 patients with moderate-to-severe OSA for 4 years and found that the stimulation threshold of the hypoglossal nerve remained constant (37). Further studies are needed to determine if this phenomenon occurs during the treatment of adolescents.

The voltage threshold of HNS is also an important factor to consider and to investigate. In adolescents, the voltage threshold for HNS may differ from that of adults. Since adolescents with DS are going through a particular stage of rapid physical growth, the efficacy and safety of HNS treatment should be focused on. While four studies have been examined at titrating stimulation voltage, none have precisely investigated how variations in voltage stimulation intensity affect the efficacy of the HNS treatment. Diercks et al. observed that increasing stimulation voltage during HNS treatment significantly reduced the AHI of patients (15). The threshold of voltage stimulation required for adolescents did not seem to change with age.

So far, it is unknown whether higher voltage stimulation is necessary during HNS therapy to obtain improved efficacy. According to certain studies, HNS can effectively control OSA in adolescents with DS, but the underlying OSA is likely to continue until adulthood (23). Therefore, a longer period of follow-up is required to evaluate the long-term effectiveness of HNS for adolescents with DS and OSA.

It is well recognized that adolescents with DS suffer from cognitive impairment and that prolonged sleep hypopnea might worsen this impairment (38). Furthermore, adolescents are still in a crucial stage of intellectual and cognitive development; and thus, they may benefit from early OSA treatment. A study of neurocognition and behavior in nine adolescents treated with HNS found that these participants had better neurocognitive and behavioral scores after 6.5 months of treatment for an average of 15.2 ± 3.4 h per day (22). The actual treatment of adolescents requires more effort from their families daily. Nevertheless, HNS therapy can effectively reduce the burden on families.

BMI may have a significant impact on HNS treatment outcomes. The Food and Drug Administration (FDA) has recommended HNS for treating OSA in neurotypical adults with an AHI <50 events/h, a BMI <32 kg/m^2^, and no circumferential airway collapse at the level of the velopharynx (12, 39). Adolescents with DS are still in a crucial stage of BMI (40). According to one study, increased BMI during treatment may explain the necessity for voltage titration (23). Currently, no more data exists to determine how the BMI of Adolescents with DS affects the therapeutic effect of HNS.

Adolescents with DS who were treated with HNS may experience complications or adverse events. Therefore, understanding the reasons behind adverse occurrences and their consequences can help improve therapeutic outcomes. Three studies reported adverse events, such as tongue or mouth pain, rash, tissue inflammation, cheek swelling, irritation-related discomfort, insomnia, pneumothorax, and swallowing or speech-related problems. These adverse events were primarily caused by device displacement, infection, device migration, and poor postoperative pain control. If the patient has a small chest, the stimulator could become squeezed by the beating heart (41). As the patient ages and their body size increases, it is vital to evaluate whether the length of the device wires is sufficient. Therefore, we could reduce complications by selecting an appropriate surgical site and a matched electrical stimulation device, avoiding migration of the device and lead requires adequate anchoring and limited sac dissection during device placement, and reducing oral discomfort or pain by titrating the voltage. Fortunately, no permanent injuries, life-threatening illnesses, or deaths have been reported in the literature.

This review has some limitations. First, the sample sizes of the included studies were limited, and several were case reports. Also, none of these studies had a control group. Thus, these investigations might be affected by research bias. Second, the safety of HNS therapy and the reasons behind some adverse events, including some severe ones, weren't fully understood by these investigations. Additionally, there was no record of adolescent tolerance to electrical stimulation in these investigations. It is important to consider the safety of HNS therapy and guard against adverse outcomes. Third, the follow-up duration of these participants was typically limited, and the long-term consequences of HNS on adolescents with DS and OSA remains unknown. Therefore, large-scale, prospective, randomized controlled, multicenter studies, are required in the future.

## Conclusions

In conclusion, HNS can significantly reduce the AHI and improve the quality of life of adolescents with DS, and can be considered as a potential alternative treatment for OSA. As adolescents get older, more studies are required to fully demonstrate the effectiveness of HNS, with a greater focus on potential complications, adverse events, long-term efficacy, comfort, and cost-effectiveness throughout HNS treatment. Comprehensive therapy protocols incorporating two or more therapeutic techniques, including CPAP, upper airway surgery, and HNS, are also worthy of investigation. Currently, HNS has not yet received FDA approval for pediatric patients, which has restricted its widespread clinical application.

## Data availability statement

The original contributions presented in the study are included in the article/Supplementary material, further inquiries can be directed to the corresponding authors.

## Author contributions

PL: conceptualization, methodology, writing, review and editing, and approval for final version. WK and KZ: investigation, data curation, formal analysis, and approval for final version. CF: investigation, data curation, software, and approval for final version. XD: methodology, writing—review and editing, and approval for final version. XM: conceptualization, writing, review and editing, and approval for final version. All authors contributed to the article and approved the submitted version.

## Conflict of interest

The authors declare that the research was conducted in the absence of any commercial or financial relationships that could be construed as a potential conflict of interest.

## Publisher's note

All claims expressed in this article are solely those of the authors and do not necessarily represent those of their affiliated organizations, or those of the publisher, the editors and the reviewers. Any product that may be evaluated in this article, or claim that may be made by its manufacturer, is not guaranteed or endorsed by the publisher.
