# Supplementary material for: Hypoglossal nerve stimulation in adolescents with down syndrome and obstructive sleep apnea: A systematic review and meta-analysis
Source: Front Neurol. 2022 Oct 25;13:1037926. doi: 10.3389/fneur.2022.1037926 (PMC9640576; doi:10.3389/fneur.2022.1037926)
Supplement: Supplementary file 1 [file Data_Sheet_1.docx]

**Search Strategy for the Systematic Review**

1. **PubMed (n = 16)**

Search strategy:

(((((((((((((((Down Syndrome) OR (Syndrome, Down)) OR (Mongolism)) OR (47,XY,+21)) OR (Trisomy G)) OR (47,XX,+21)) OR (Down's Syndrome)) OR (Downs Syndrome)) OR (Syndrome, Down's)) OR (Trisomy 21)) OR (Trisomy 21, Mitotic Nondisjunction)) OR (Down Syndrome, Partial Trisomy 21)) OR (Partial Trisomy 21 Down Syndrome)) OR (Trisomy 21, Meiotic Nondisjunction)) AND ((((((((((((((((((((((((Obstructive Sleep Apnea,) OR (Sleep Apnea Syndromes)) OR (Apnea Syndrome, Sleep)) OR (Apnea Syndromes, Sleep)) OR (Sleep Apnea Syndrome)) OR (Sleep Hypopnea)) OR (Hypopnea, Sleep)) OR (Hypopneas, Sleep)) OR (Sleep Hypopneas)) OR (Apnea, Sleep)) OR (Apneas, Sleep)) ) OR (Sleep Apnea)) OR (Sleep Apneas)) OR (Sleep Apnea, Mixed Central and Obstructive)) OR (Mixed Central and Obstructive Sleep Apnea)) OR (Sleep Apnea, Mixed)) OR (Mixed Sleep Apnea)) OR (Mixed Sleep Apneas)) OR (Sleep Apneas, Mixed)) OR (Hypersomnia with Periodic Respiration)) OR (Sleep-Disordered Breathing)) OR (Breathing, Sleep-Disordered)) OR (Sleep Disordered Breathing))) AND ((((((((Hypoglossal nerve stimulation) OR (Electrical Stimulation)) OR (Electrical Stimulations)) OR (Stimulation, Electrical)) OR (Stimulations, Electrical)) OR (Stimulation, Electric)) OR (Electric Stimulations)) OR (Stimulations, Electric))

1. **Web of science (n = 23)**

Search strategy:

#1 TS=(Down Syndrome) OR TS=(Down Syndrome) OR TS=(Syndrome, Down) OR TS=(Mongolism) OR TS=(47,XY,+21) OR TS=(Trisomy G) OR TS=(47,XX,+21) OR TS=(Down's Syndrome) OR TS=(Downs Syndrome) OR TS=(Syndrome, Down's) OR TS=(Trisomy 21) OR TS=(Trisomy 21, Mitotic Nondisjunction) OR TS=(Down Syndrome, Partial Trisomy 21) OR TS=(Partial Trisomy 21 Down Syndrome) OR TS=(Trisomy 21, Meiotic Nondisjunction)

#2 TS=(Sleep Apnea Syndromes) OR TS=(Sleep Apnea Syndromes) OR TS=(Apnea Syndrome, Sleep) OR TS=(Apnea Syndromes, Sleep) OR TS=(Sleep Apnea Syndrome) OR TS=(Sleep Hypopnea) OR TS=(Hypopnea, Sleep) OR TS=(Hypopneas, Sleep) OR TS=(Sleep Hypopneas) OR TS=(Apnea, Sleep) OR TS=(Apneas, Sleep) OR TS=(Sleep Apnea) OR TS=(Sleep Apneas) OR TS=(Sleep Apnea, Mixed Central and Obstructive) OR TS=(Mixed Central and Obstructive Sleep Apnea) OR TS=(Sleep Apnea, Mixed) OR TS=(Mixed Sleep Apnea) OR TS=(Mixed Sleep Apneas) OR TS=(Sleep Apneas, Mixed) OR TS=(Hypersomnia with Periodic Respiration) OR TS=(Sleep-Disordered Breathing) OR TS=(Breathing, Sleep-Disordered) OR TS=(Sleep Disordered Breathing)

#3 TS=(Hypoglossal nerve stimulation) OR TS=(Hypoglossal nerve stimulation) OR TS=(Upper airway stimulation) OR TS=(Hypoglossal Nerve Stimulator )

#4 #1 AND #2 AND #3

1. **Embase (n = 24)**

Search strategy:

#1 'down syndrome'/exp OR 'down syndrome' OR (down AND ('syndrome'/exp OR syndrome)) OR 'syndrome, down':ab,ti OR mongolism:ab,ti OR 47,xy,+21:ab,ti OR 'trisomy g':ab,ti OR 47,xx,+21:ab,ti OR 'down syndrome':ab,ti OR 'downs syndrome':ab,ti OR 'syndrome, downs':ab,ti OR 'trisomy 21':ab,ti OR 'trisomy 21, mitotic nondisjunction':ab,ti OR 'down syndrome, partial trisomy 21':ab,ti OR 'partial trisomy 21 down syndrome':ab,ti OR 'trisomy 21, meiotic nondisjunction':ab,ti**76326**

#2 'sleep apnea syndromes'/exp OR 'sleep apnea syndromes' OR (('sleep'/exp OR sleep) AND ('apnea'/exp OR apnea) AND syndromes) OR 'apnea syndrome, sleep':ab,ti OR 'apnea syndromes, sleep':ab,ti OR 'sleep apnea syndrome':ab,ti OR 'sleep hypopnea':ab,ti OR 'hypopnea, sleep':ab,ti OR 'hypopneas, sleep':ab,ti OR 'sleep hypopneas':ab,ti OR 'apnea, sleep':ab,ti OR 'apneas, sleep':ab,ti OR 'sleep apnea':ab,ti OR 'sleep apneas':ab,ti OR ('sleep apnea, mixed central':ab,ti AND obstructive:ab,ti) OR ('mixed central':ab,ti AND 'obstructive sleep apnea':ab,ti) OR 'sleep apnea, mixed':ab,ti OR 'mixed sleep apnea':ab,ti OR 'mixed sleep apneas':ab,ti OR 'sleep apneas, mixed':ab,ti OR 'hypersomnia with periodic respiration':ab,ti OR 'sleep-disordered breathing':ab,ti OR 'breathing, sleep-disordered':ab,ti OR 'sleep disordered breathing':ab,ti**99985**

#3 'hypoglossal nerve stimulation'/exp OR 'hypoglossal nerve stimulation' OR (hypoglossal AND ('nerve'/exp OR nerve) AND ('stimulation'/exp OR stimulation)) OR 'upper airway stimulation':ab,ti OR 'hypoglossal nerve stimulator':ab,ti1635

#4 #3 AND #5 AND #6 AND [25-09-2011]/sd NOT [26-06-2022]/sd24

1. **Scopus (n = 29)**

Search strategy:

**( ( ( TITLE-ABS-KEY ( down AND syndrome ) OR TITLE-ABS-KEY ( syndrome, AND down ) OR TITLE-ABS-KEY ( mongolism ) OR TITLE-ABS-KEY ( 47,xy,+21 ) OR TITLE-ABS-KEY ( trisomy AND g ) OR TITLE-ABS-KEY ( 47,xx,+21 ) OR TITLE-ABS-KEY ( down's AND syndrome ) OR TITLE-ABS-KEY ( downs AND syndrome ) OR TITLE-ABS-KEY ( syndrome, AND down's ) OR TITLE-ABS-KEY ( trisomy 21 ) OR TITLE-ABS-KEY ( trisomy AND 21, AND mitotic AND nondisjunction ) OR· TITLE-ABS-KEY ( down AND syndrome, AND partial AND trisomy 21 ) OR TITLE-ABS-KEY ( partial AND trisomy 21 down AND syndrome ) OR TITLE-ABS-KEY ( trisomy AND 21, AND meiotic AND nondisjunction ) ) ) AND ( ( TITLE-ABS-KEY ( sleep AND apnea AND syndromes ) OR TITLE-ABS-KEY ( apnea AND syndrome, AND sleep ) OR TITLE-ABS-KEY ( apnea AND syndromes, AND sleep ) OR TITLE-ABS-KEY ( sleep AND apnea AND syndrome ) OR TITLE-ABS-KEY ( sleep AND hypopnea ) OR TITLE-ABS-KEY ( hypopnea, AND sleep ) OR TITLE-ABS-KEY ( hypopneas, AND sleep ) OR TITLE-ABS-KEY ( sleep AND hypopneas ) OR TITLE-ABS-KEY ( apnea, AND sleep ) OR TITLE-ABS-KEY ( apneas, AND sleep ) OR TITLE-ABS-KEY ( sleep AND apnea ) OR TITLE-ABS-KEY ( sleep AND apneas ) OR TITLE-ABS-KEY ( sleep AND apnea, AND mixed AND central AND obstructive ) OR TITLE-ABS-KEY ( mixed AND central AND obstructive AND sleep AND apnea ) OR TITLE-ABS-KEY ( sleep AND apnea, AND mixed ) OR TITLE-ABS-KEY ( mixed AND sleep AND apnea ) OR TITLE-ABS-KEY ( mixed AND sleep AND apneas ) OR TITLE-ABS-KEY ( sleep AND apneas, AND mixed ) OR TITLE-ABS-KEY ( hypersomnia AND with AND periodic AND respiration ) OR TITLE-ABS-KEY ( sleep-disordered AND breathing ) OR TITLE-ABS-KEY ( breathing, AND sleep-disordered ) OR TITLE-ABS-KEY ( sleep AND disordered AND breathing ) ) ) ) AND ( ( TITLE-ABS-KEY ( hypoglossal AND nerve AND stimulation ) OR TITLE-ABS-KEY ( upper AND airway AND stimulation ) OR TITLE-ABS-KEY ( hypoglossal AND nerve AND stimulator ) ) )**
